# Posterior Reversible Encephalopathy in a Patient with Severe Leptospirosis Complicated with Pulmonary Haemorrhage, Myocarditis, and Acute Kidney Injury

**DOI:** 10.1155/2019/6498315

**Published:** 2019-12-17

**Authors:** W. D. D. Priyankara, E. M. Manoj

**Affiliations:** Consultant Intensivist, National Hospital of Sri Lanka, Colombo, Sri Lanka

## Abstract

Severe leptospirosis (Weil's disease) can give rise to multiorgan failure such as acute renal failure, liver dysfunction, coagulopathy, acute respiratory distress syndrome, pulmonary haemorrhage, and myocarditis. Leptospirosis is a biphasic disease characterised by leptospiraemic phase and immunological phase. Although neurological manifestations are rare in leptospirosis, aseptic meningitis, myeloradiculopathy, transverse myelitis, and cerebellar syndrome are well recognised. We report a rare case of posterior reversible encephalopathy syndrome (PRES) in a patient with severe leptospirosis during recovery phase of the illness.

## 1. Introduction

Severe leptospirosis is a life-threatening condition which can lead to multiorgan dysfunction. Various neurological manifestations such as aseptic meningitis, myeloradiculopathy, transverse myelitis, and cerebellar syndrome have been described in severe leptospirosis. We report a rare case of posterior reversible encephalopathy syndrome (PRES) in a patient with severe leptospirosis.

## 2. Case Report

**Figure 1:**
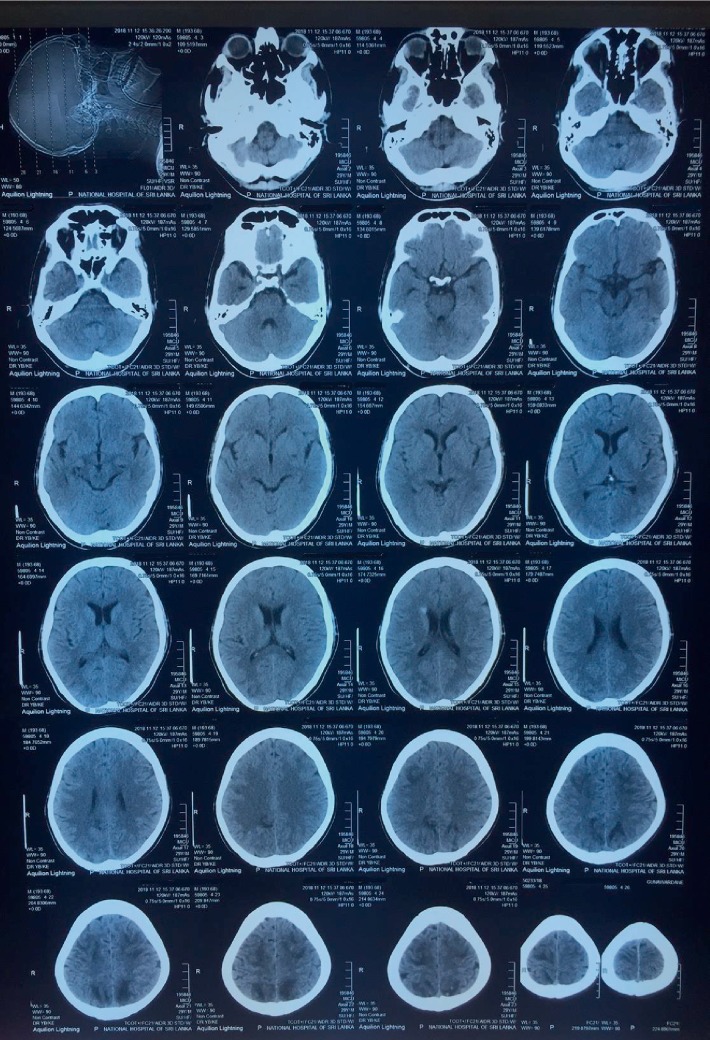
Noncontrast computerised tomography showing hypodense areas involving bilateral occipital regions.

**Figure 2:**
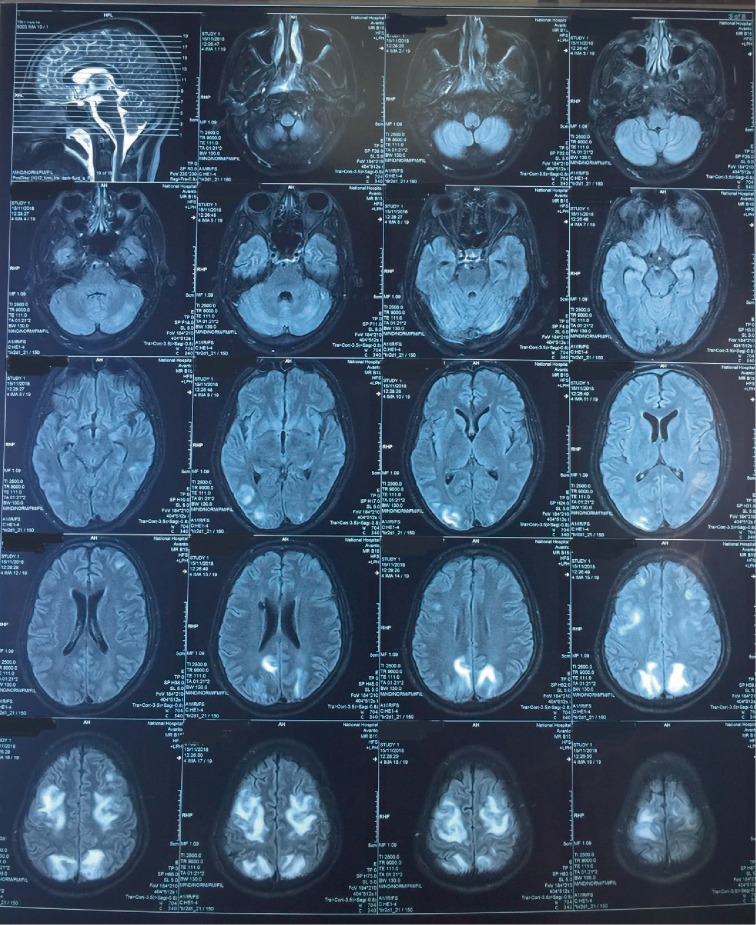
Magnetic resonant imaging showing bilaterally symmetrical parieto-occipital white matter hyperintensities.

A 29-year-old manual labourer was admitted to the hospital with a 4-day history of fever, myalgia, and reduced urine output. He was previously healthy without known long-term medical conditions. However, he was addicted to heroin. On admission to the ward, he was febrile (102°F), icteric, and he was complaining of severe myalgia. His vital parameters were stable with a heart rate of 110 b/min, blood pressure of 100/60 mmHg, and pulse oximeter saturation reading of 96% on room air. His white cell count was raised (13.1 × 10^9^/L) with neutrophil leucocytosis and the platelet count was 31 × 10^9^/L. His C-reactive protein was 234 mg/L. He was oliguric with a serum creatinine of 189 *µ*mol/L. His ECG revealed anterior T wave inversion with sinus tachycardia and a raised troponin I of 3 ng/ml (control = <0.5) suggestive of myocarditis. He was managed as severe leptospirosis and treated with intravenous cefotaxime. The next day he developed hypotension, haemoptysis, and worsening renal failure with anuria and rising creating of 245 *µ*mol/L necessitating intensive care admission. On admission to the ICU, his heart rate was 115 b/min with a blood pressure of 90/40 (57) mmHg. Bed side echocardiogram showed mild left ventricular dysfunction. He was tachypnoeic with a respiratory rate of 32 breaths/min and received supplementary oxygen to maintain arterial saturation of >92% and PaO2 of >65 mmHg. His chest X-ray showed bilateral alveolar shadows which was suggestive of pulmonary haemorrhage.His coagulation profile was normal with APTT of 28 and INR of 1. He was managed with judicious fluids resuscitation and noradrenaline via a central venous catheter to maintain mean arterial pressure of more than 65 mmHg. He received 3 consecutive cycles of plasma exchange and 3 days of 1 g of methylprednisolone for pulmonary haemorrhage. Furthermore, he required 2 sessions of slow efficiency haemodialysis for his acute kidney injury. His Leptospira microagglutination test was positive for *Leptospira bakeri* with a titre >1/1240 on day 14 of the illness.He made a gradual improvement over the next 5 days and he was weaned from oxygen and vasopressor therapy gradually. On the sixth day in the ICU, he became confused, agitated, and developed refractory generalised tonic clonic seizures with a rise in blood pressure (ranging from 140/ 100 mmHg to 160/120 mmHg) requiring intubation and mechanical ventilation. Heroin withdrawal and lepto-meningitis were considered as the initial differential diagnoses for his seizures. He was sedated with intravenous midazolam and started on Levetiracetam for the seizures. His noncontrast tomography (CT) scan (Figure 1) showed hypodense lesions involving both occipital regions raising the possibility of PRES, which was confirmed by a magnetic resolution imaging (MRI) (Figure 2). We have not performed a lumbar puncture as his platelet counts remained low. He was started on intravenous labetalol to maintain SBP between 110 and 120 mmHg. His neurology gradually improved over the next 24–48 hours and he was extubated successfully after four days. He was discharged to the ward successfully after 15 days in the ICU.

## 3. Discussion

Leptospirosis is a zoonosis caused by the spirochete *Leptospira interrorgan*. Severe leptospirosis (Weil's disease) can give rise to multiorgan failure such as acute renal failure, liver dysfunction, coagulopathy, acute respiratory distress syndrome, pulmonary haemorrhage, and myocarditis [1]. Leptospirosis is a biphasic disease. First phase (leptospiraemic phase) is characterised by fever, myalgia, headache, conjunctival suffusion, and other nonspecific constitutional symptoms. Second phase (Immune phase) is characterized by organ dysfunction which could lead to death [1].

The incidence of leptospirosis and the morbidity and mortality is high in regions of south and southeast Asia [2]. In Sri Lanka, there were more than 5000 cases reported during the period between 2008 and 2014 with a case fatality rate of 1-2% [3, 4].

Aseptic meningitis is the commonest neurological manifestation of leptospirosis [5]. The other neurological manifestations include, myeloradiculopathy, transverse myelitis, and cerebellar syndrome. However, these clinical syndromes are rare [5]. Aram J reported a case of PRES in a patient with leptospirosis and the patient had clinical features of PRES after three weeks of antibiotic therapy [6]. This was postulated to have occurred due to immune-mediated endothelial damage leading to vasogenic oedema. However, our patient had PRES early in the immune phase.

PRES is a clinical syndrome characterised by headache, altered level of consciousness, visual changes, and seizures, which is associated with characteristic findings of posterior cerebral white matter oedema on neurological imaging [7]. This syndrome is well described in systemic vasculitides such as systemic lupus erythematosus, polyarteritis nodosa, and cryoglobulinemia [7]. Even though the condition is reversible in most cases, as suggested by the name, early recognition and treatment is crucial to prevent permanent brain damage. Cerebral vasoconstriction, failure of cerebral autoregulation with vasogenic oedema, and endothelial damage with leakage of fluid in the brain are postulated as the reasons for the pathogenesis of PRES [8]. Predilection for the posterior circulation is thought to be due to lack of sympathetic innervation of the arterioles of the vertebro-basilar system compared to the anterior circulation of the brain [7].

Severe leptospirosis is thought to be a form of systemic vasculitis. De Brito and colleagues demonstrated coronary arteritis and aortitis in autopsies of patients who died of severe leptospirosis [9]. Furthermore, they suggested that toxins, and various antigens released by the lysis of Leptospira might lead to the injury of the endothelium of capillaries. However, the mechanism of vascular damage is not completely known. The transient hypertension seen in our patient at the recovery phase would have precipitated an endothelial leak causing vascular injury in the cerebral vasculature leading to PRES. Careful management of his blood pressure with intravenous labetalol resulted in a marked improvement of his neurological manifestations.

## 4. Conclusion

Severe leptospirosis can lead to multiorgan dysfunction and it has a high mortality rate. Neurological manifestations are recognised manifestations in severe leptospirosis. However, there is only a single case of PRES reported due to leptospirosis. Our case highlights the importance of having a high degree of suspicion of PRES in patients who develop seizures in the immunological phase of leptospirosis. Careful management of the blood pressure will lead to favourable outcomes in this condition.
